# Beyond bronchitis: a review of the congenital and acquired abnormalities of the bronchus

**DOI:** 10.1007/s13244-016-0537-y

**Published:** 2016-12-13

**Authors:** Thomas Marini, Susan K. Hobbs, Abhishek Chaturvedi, Kathrine Kaproth-Joslin

**Affiliations:** Department of Imaging Sciences, University of Rochester Medical Center, 601 Elmwood Ave, Box 648, Rochester, NY 14642 USA

**Keywords:** Bronchus, Bronchial stenosis or obstruction, Bronchial inflammation, Bronchial infection, Bronchiectasis

## Abstract

Anomalies of the bronchus can be both congenital and acquired. Several different congenital aberrations of the bronchial anatomy are commonly encountered including tracheal bronchus, accessory cardiac bronchus, and bronchial agenesis/aplasia/hypoplasia. In addition, Williams-Campbell syndrome and cystic fibrosis are two other congenital conditions that result in bronchial pathology. Acquired pathology affecting the bronchi can typically be divided into three broad categories of bronchial disease: bronchial wall thickening, dilatation/bronchiectasis, and obstruction/stenosis. Bronchial wall thickening is the common final response of the airways to irritants, which cause the bronchi to become swollen and inflamed. Bronchiectasis/bronchial dilatation can develop in response to many aetiologies, including acquired conditions such as infection, pulmonary fibrosis, recurrent or chronic aspiration, as well as because of congenital conditions such as cystic fibrosis. The causes of obstruction and stenosis are varied and include foreign body aspiration, acute aspiration, tracheobronchomalacia, excessive dynamic airway collapse, neoplasm, granulomatous disease, broncholithiasis, and asthma. Knowledge of normal bronchial anatomy and its congenital variants is essential for any practicing radiologist. It is the role of the radiologist to identify common imaging patterns associated with the various categories of bronchial disease and provide the ordering clinician a useful differential diagnosis tailored to the patient’s clinical history and imaging findings.

*Teaching Points*

• *Bronchial disorders are both congenital and acquired in aetiology*.

• *Bronchial disease can be divided by imaging appearance*: *wall thickening*, *dilatation*, *or obstruction*.

• *Bronchial wall thickening is the common final response of the airways to irritants*.

• *Imaging patterns must be recognised and the differential diagnosis tailored for patient management*.

## Introduction

**Fig. 1:**
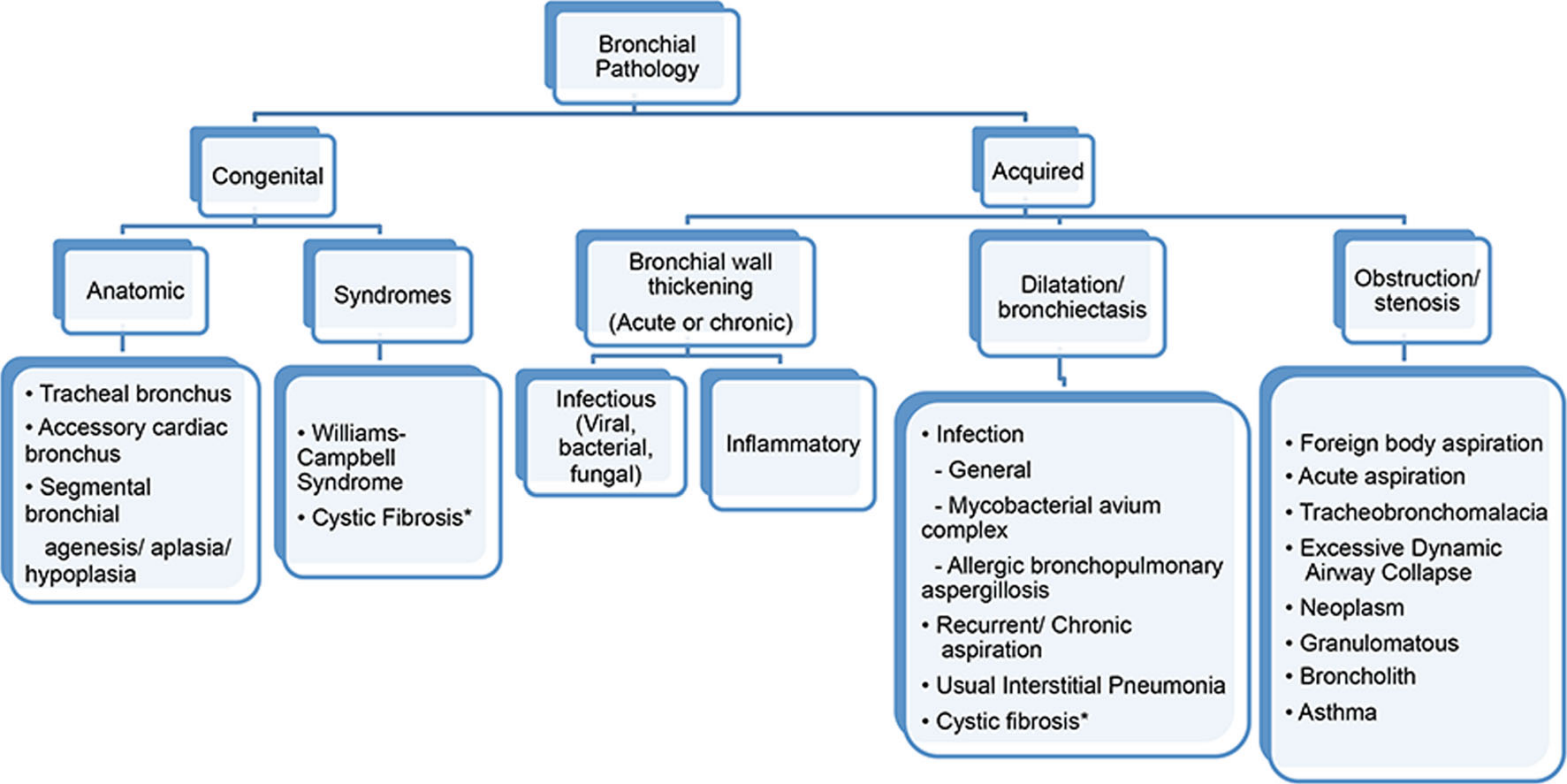
Conceptual organisation of bronchial pathology

The bronchi are the main branching components of the conduction zone in the respiratory system serving as the anatomical bridge between the trachea and the bronchioles. Anomalies of the bronchus can be both congenital and acquired. While congenital bronchial abnormalities are often secondary to anatomic variation, acquired bronchial disorders as well as some congenital conditions can typically be divided into three broad categories based on imaging findings: bronchial wall thickening, dilatation/bronchiectasis, and obstruction/stenosis [1]. Regardless of the underlying aetiology, the clinical presentation of bronchial disease tends to be non-specific with complaints typically including cough, wheezing, and shortness of breath. Patients with both acute and long-standing symptoms of bronchial disease often undergo diagnostic imaging for further evaluation, making awareness of this topic critical. In addition, some forms of bronchial pathology are asymptomatic or minimally symptomatic and are discovered incidentally on imaging performed for other indications. A schematic review of bronchial pathology is presented in Fig. 1.

## Overview of diagnostic imaging techniques in evaluating bronchial disease

The radiologist has a wide array of imaging modalities to evaluate for bronchial disease. The initial study of choice in virtually all cases of bronchial disease is the chest standard frontal and lateral x-ray for its ease and convenience [2]. If further investigation is necessary, computed tomography (CT) is typically the next imaging modality of choice [3]. For these studies, contrast administration is often not necessary. Thin axial slices are recommended to allow for isotropic imaging and improved post-processing, including two-dimensional multiplanar reformatting, three-dimensional volume rendering, and virtual endoscopy, as these can be important diagnostic tools to better understand the anatomy of the bronchi and elucidate pathology [4]. Specialised imaging, such as high-resolution chest CT (HRCT), can be useful for assessment of conditions such as pulmonary fibrosis and bronchiectasis [5]. Occasionally, dynamic imaging is used to evaluate for conditions such as tracheobronchomalacia and Williams-Campbell syndrome when abnormalities may only be present during certain phases of the respiratory cycle [6]. Scanning children (especially those under 5 years of age) can be challenging because of an inability to follow technologist instructions, sometimes requiring sedation and intubation for scanning; however many of the newer multidetector CTs have faster scan times and can decrease or eliminate the need for sedation and/or intubation [7].

## Congenital conditions

### Tracheal bronchus

**Fig. 2:**
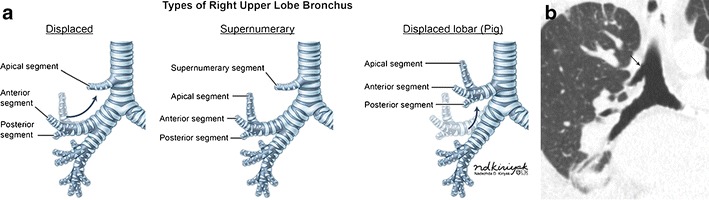
Tracheal bronchus. **a** Illustrations of the tracheal bronchi types. **b** Coronal CT imaging demonstrates a supernumerary right upper lobe bronchus (*solid arrow*) originating from the trachea

This term describes an aberrant or accessory bronchus supplying the upper lobe originating from the lateral wall of the trachea, with some definitions also including branches originating from the mainstem bronchi or carina. It is more commonly right-sided, within 2 cm of the carina, and of variable length (sometimes blind ending) [8, 9]. There are two common tracheal bronchus types: supernumerary and displaced [8, 9]. A supernumerary tracheal bronchus exists in addition to an anatomically normal branching upper lobe bronchus (Fig. 2a and b). A displaced bronchus (more common) occurs when one segmental branch of the anatomically normal upper lobe bronchus is simultaneously absent and “replaced” by an aberrant bronchus originating from the trachea (Fig. 2a). The term “pig bronchus” or “bronchus suis” is used when there is tracheal origin of the entire right upper lobe bronchial system (Fig. 2a). Tracheal bronchi are usually asymptomatic however can present with recurrent infections, atelectasis, and bronchiectasis, especially when blind ending.

### Accessory cardiac bronchus

**Fig. 3:**
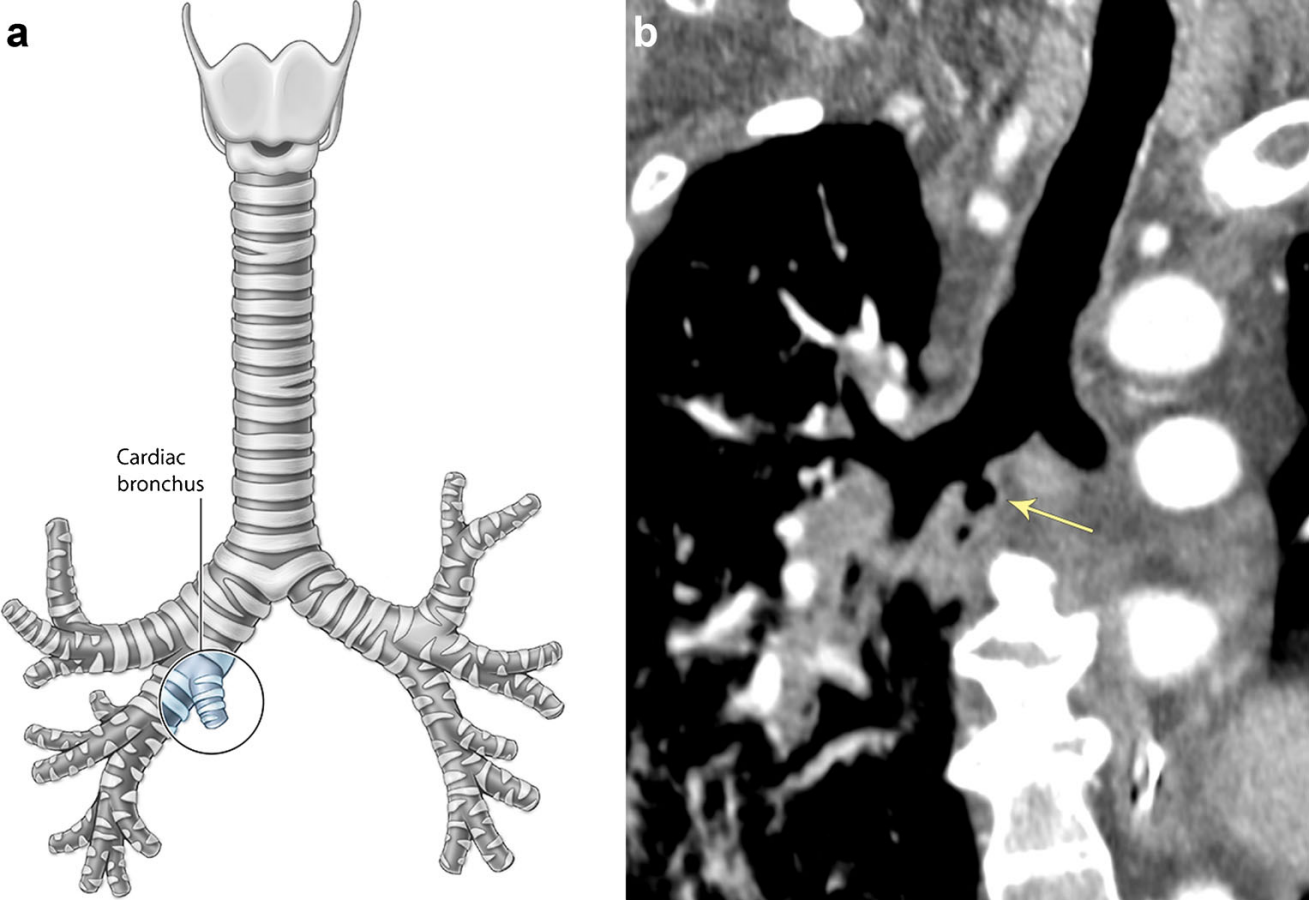
Accessory cardiac bronchus. **a** Illustration of a cardiac bronchus arising from the right main bronchus. **b** Coronal CT imaging of an accessory cardiac bronchus (*arrow*) arising from the right main bronchus

Located in the azygo-esophageal recess, this rare anatomical variant describes an accessory bronchus originating from the medial wall of the right or left main bronchus or bronchus intermedius (Fig. 3a and b) [8, 10]. It is typically blind ending but can occasionally branch and terminate in a small portion of ventilated normal lung, hypoplastic lung, or an area of cystic degeneration separated by an accessory fissure [8]. The condition is often asymptomatic however may present with recurrent infection, atelectasis, and haemoptysis.

### Bronchial agenesis/aplasia/hypoplasia

**Fig. 4:**
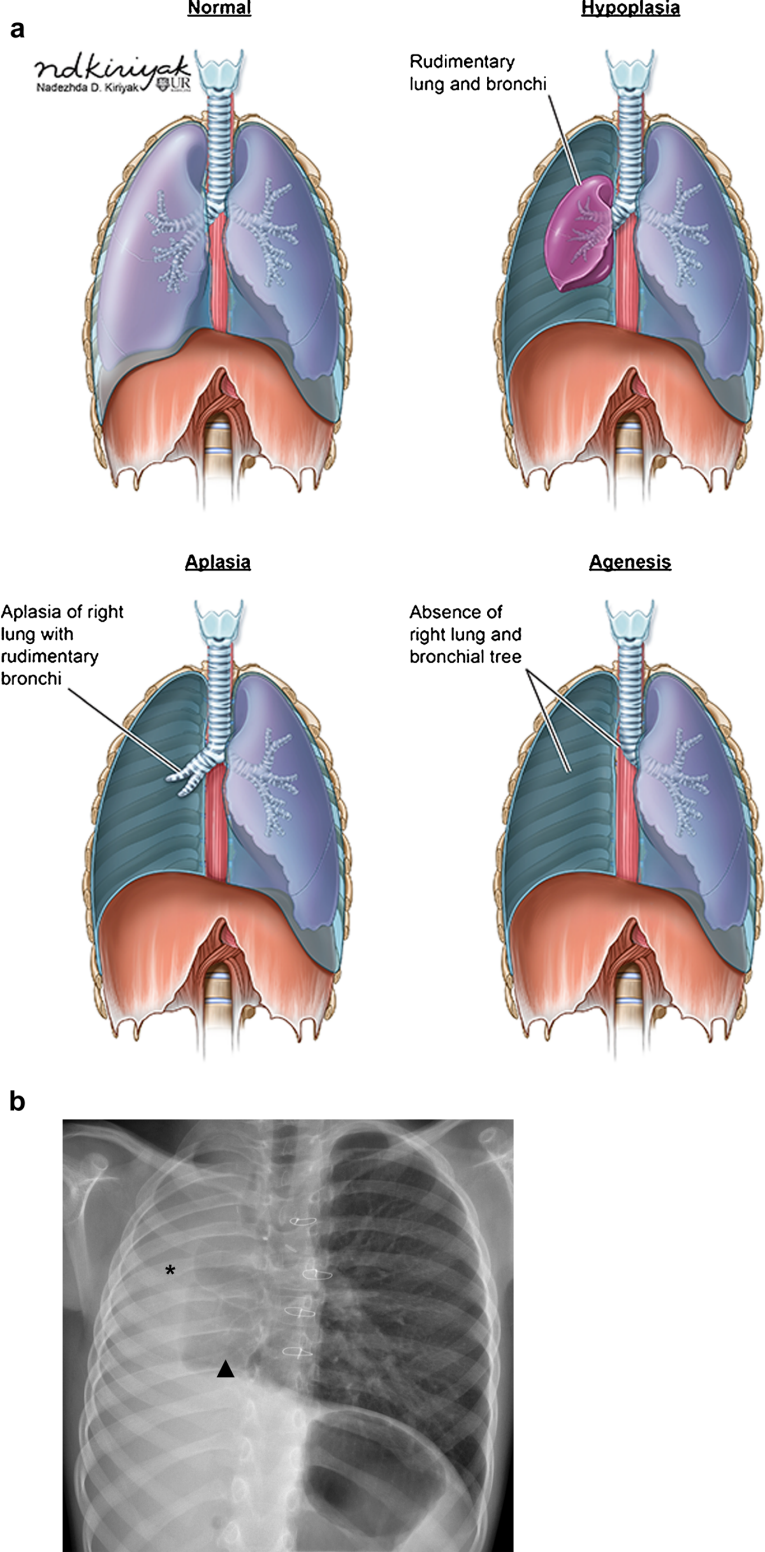
Congenital pulmonary and bronchial malformation. **a** Illustrations demonstrating lobar bronchial malformations resulting in agenesis, aplasia, or hypoplasia as compared to normal. **b** Frontal chest x-ray demonstrating agenesis of the right mainstem bronchus and right lung (*); note the hyperexpanded left lung crossing the midline (*triangle*)

These terms refer to a spectrum of congenital pulmonary malformations with either absent or rudimentary development of the segmental or lobar bronchus and associated pulmonary parenchyma (Fig. 4a and b) [11, 12]. Bronchial agenesis is complete absence of a bronchus and its associated lung (Fig. 4a and b). In bronchial aplasia, a rudimentary bronchus is present, but there is complete absence of the associated lung parenchyma (Fig. 4a). Bronchial hypoplasia refers to the presence of a small/rudimentary bronchus with variable amounts of lung tissue (Fig. 4a).

### Williams-Campbell syndrome

**Fig. 5:**
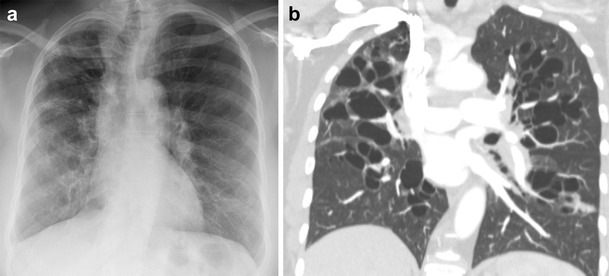
Williams-Campbell syndrome. **a** Frontal chest x-ray demonstrating bilateral architectural distortion and coarse interstitial markings with cystic appearing regions in the lower lobe consistent with the scarring and diffuse saccular bronchiectasis seen in Williams-Campbell syndrome. **b** Coronal CT imaging demonstrating the severe bilateral cystic bronchiectasis of the subsegmental (4th-6th generation) bronchi commonly seen in Williams-Campbell syndrome

This congenital condition occurs because of a cartilage abnormality involving the 4th-6th order subsegmental bronchi, resulting in severe bronchiectasis and recurrent pulmonary infections. The condition typically presents in childhood and infancy with symptoms of coughing, wheezing, and dyspnoea. Imaging reveals normal central airways with severe bilateral cystic bronchiectasis in the subsegmental bronchi, often associated with bronchial wall thickening, mucous plugging, and bronchomalacia (Fig. 5a and b) [13]. During dynamic imaging, the abnormal bronchi will demonstrate ballooning on inspiratory imaging and collapse/air-trapping on expiratory imaging [14].

### Cystic fibrosis

To be covered in the acquired/bronchiectasis section.

## Acquired conditions

### Bronchial wall thickening

#### Bronchitis

Bronchitis is the generic term referring to inflammation of the bronchial wall, representing the common final response of the airways to irritants. Acute bronchitis is a short-term process (<3 months in length but typically lasting 2–10 days) with symptoms occasionally lingering for 2–3 weeks post-infection, most commonly triggered by a viral upper respiratory infection leading to an inflammatory hyperresponsiveness [15]. Patients typically present with cough, wheezing, dyspnoea, chest discomfort, fever, and occasionally sputum production. Chronic bronchitis is defined as a productive cough most days for ≥3 months in 2 consecutive years in patients for whom other causes of chronic cough have been excluded [16].

**Fig. 6:**
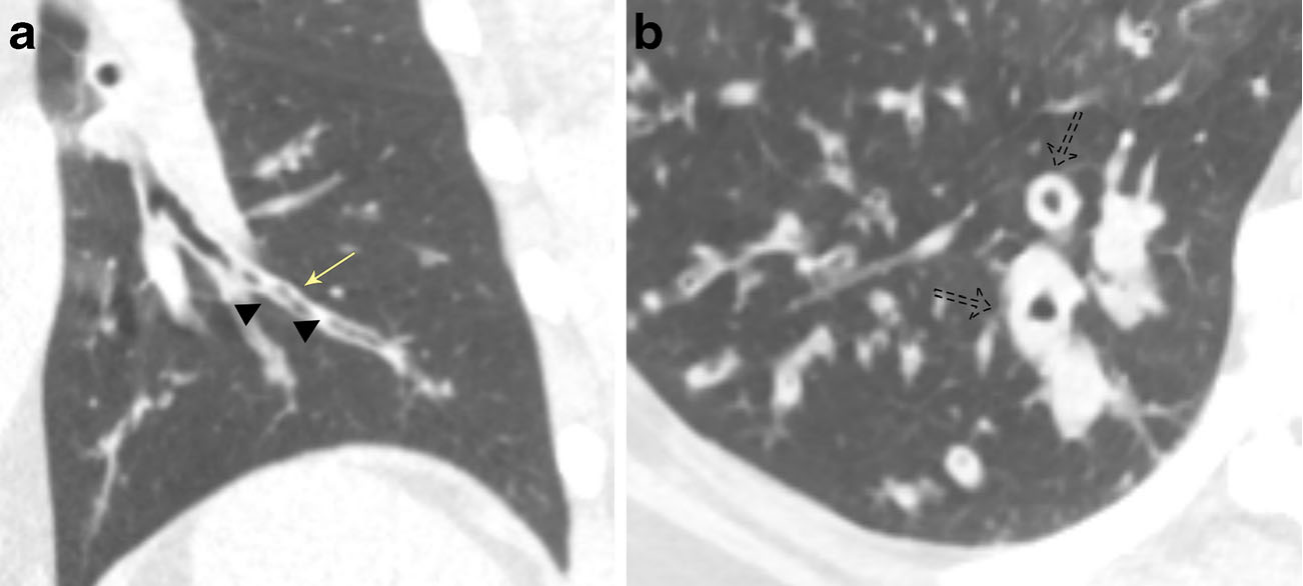
Bronchial wall thickening due to bronchitis. Coronal (**a**) and axial (**b**) CT imaging demonstrates bronchial wall thickening and areas of mucous plugging (*arrowhead*). Tram tracking refers to wall thickening in longitudinally oriented bronchi (*solid arrow*) mimicking the parallel rail tracks of a tram whereas peribronchial cuffing (*dashed arrow*) refers to wall thickening of the bronchi seen in cross section, sometimes referred to as the “donut sign”

In both acute and chronic bronchitis, the chest x-ray is often unremarkable. CT demonstrates bronchial wall thickening, a mosaic attenuation pattern of the pulmonary parenchyma, and mucous plugging (Fig. 6a and b) [17]. The descriptive terms “tram tracking” and “peribronchial cuffing” are often used to describe bronchial wall thickening, with the former describing thickened longitudinally oriented bronchi mimicking the parallel rail tracks of a tram and the latter referring to thickened bronchi seen in cross section, also called the “donut sign” (Fig. 6a and b) [18, 19].

### Dilatation/bronchiectasis

#### Bronchiectasis overview

**Fig. 7:**
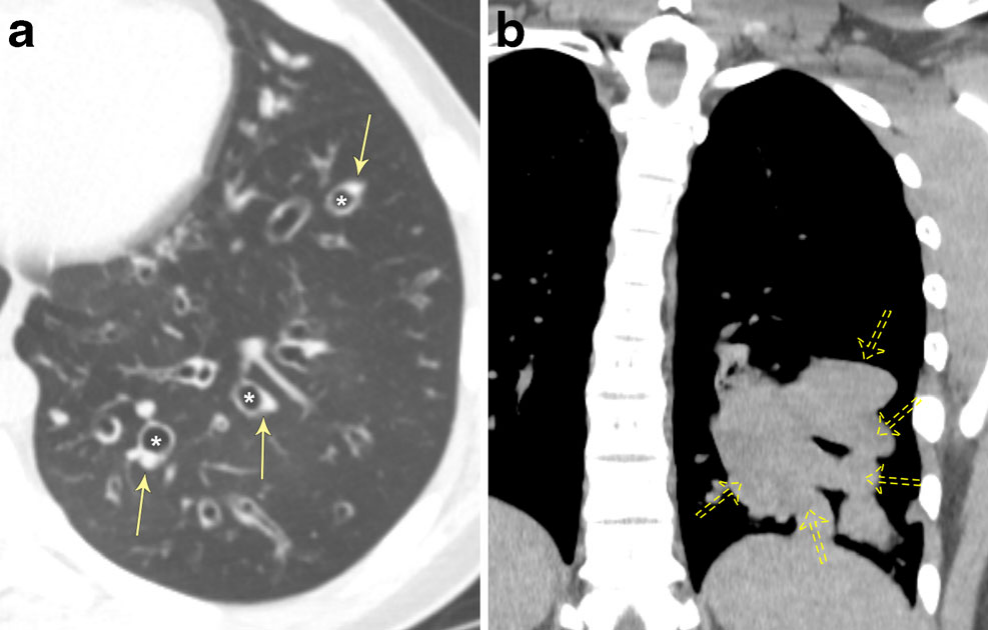
Classic imaging signs of bronchiectasis. **a** Signet ring sign is a CT finding referring to a markedly enlarged bronchus (*) mimicking a ring and its adjacent normal accompanying pulmonary artery representing the signet emblem of the ring (*arrow*). **b** Finger-in-glove sign describes the presence of mucous or fluid impaction within a dilated bronchus (*dashed arrow*) appearing as a radiopaque “finger” in the “glove” of the bronchus

Bronchiectasis is the irreversible dilatation of a bronchus. The normal bronchial diameter is typically between 0.7 and 1 times the adjacent pulmonary artery branch diameter, and diameters greater than 1.5 times are considered dilated [20]. Other signs of bronchiectasis include failure to taper as the bronchus courses peripherally and visualisation of the bronchus within 1 cm of the pleural surface [21]. Bronchial dilatation should be present for >6 months to establish its chronicity. Two classic signs of bronchiectasis include the signet ring sign and the finger-in-glove sign (Fig. 7a and b). The signet ring sign, visible on CT imaging, describes a markedly enlarged bronchus mimicking a ring with its accompanying normal pulmonary artery representing the signet emblem (Fig. 7a) [22]. The finger-in-glove sign describes mucous impaction within a dilated bronchus appearing as a radiopaque “finger” in the “glove” of the bronchus (Fig. 7b) [23].

**Fig. 8:**
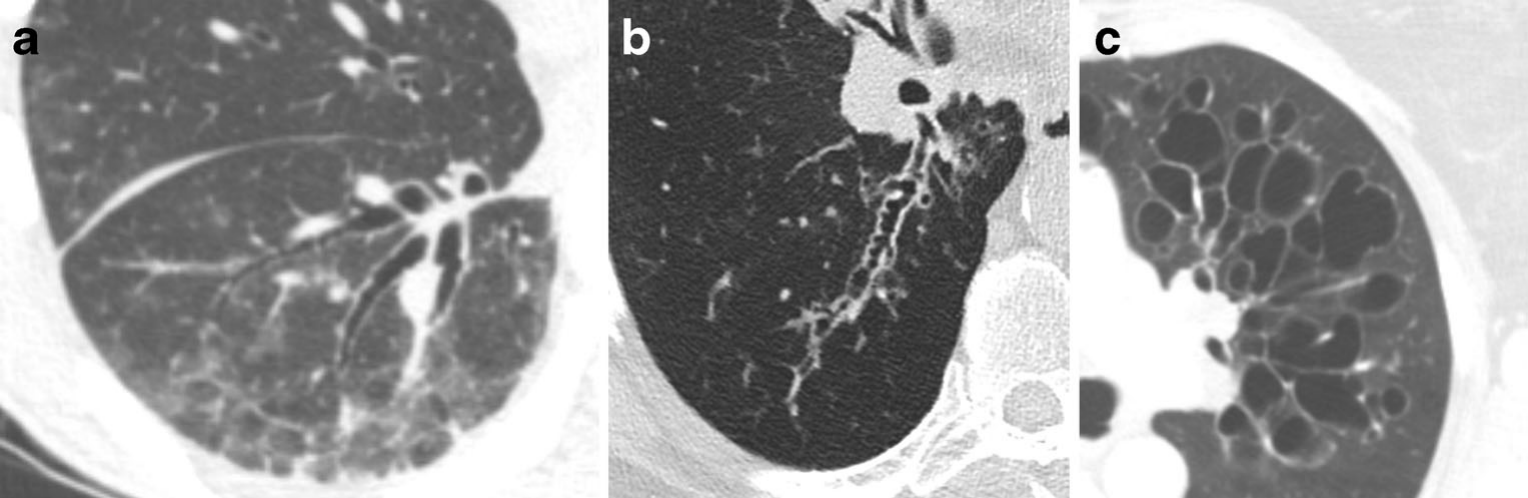
Types of bronchiectasis. Axial CT imaging of three different patients demonstrating the bronchiectasis types. **a** Cylindrical-type bronchiectasis is characterised by uniform nontapering or gradually tapering bronchial dilatation. **b** Varicose-type bronchiectasis is characterised by a beaded or “string-of-pearls” appearance with areas of alternating bronchial narrowing and dilatation. **c** Cystic-type bronchiectasis is characterised by markedly dilated cyst-like bronchi, often extending to the pleural surface with a “cluster-of-grapes” appearance

There are three main forms of bronchiectasis, characterised by morphological pattern: cylindrical, varicose, and cystic types (Fig. 8a–c) [24]. Cylindrical bronchiectasis is the most common and is characterised by bronchial dilatation with uniform nontapering or gradual tapering (Fig. 8a). Varicose bronchiectasis is less common and is characterised by a beaded or “string-of-pearls” appearance in which areas of alternating bronchial narrowing and dilatation are present (Fig. 8b). Cystic bronchiectasis is the severest and rarest form of bronchiectasis and is characterised by markedly dilated cyst-like bronchi, which often extend to the pleural surface with a “cluster-of-grapes” appearance (Fig. 8c). The three forms of bronchiectasis can be (and often are) present in the same patient, with the extent and nature of bronchial dilatation often falling along a spectrum of imaging findings.

#### Bronchiectasis in general infections

Post-infectious bronchiectasis is the most common cause of bronchiectasis, occurring secondary to viral, bacterial, and fungal aetiologies [24]. This condition should not be confused with post-infectious transient dilatation of the bronchi, a finding that can persist for up to 4–6 months after an episode of pneumonia [1]. Bronchial changes typically occur in the location of the original infectious process. Lobar bacterial pneumonia may result in focal bronchiectasis of a specific lobe whereas atypical or viral infection may cause more diffuse changes. Elucidating the exact causative organism of post-infectious bronchiectasis is typically difficult because of significant overlap in imaging findings between different infectious organisms; however there are a few pathogens that have specific patterns of bronchiectasis, which, if recognised by the imager, can assist in diagnosis and treatment outcomes. Two of these organisms commonly encountered are *Mycobacterial avium complex* (MAC) and *Aspergillosis*.

#### Bronchiectasis due to MAC Infection

**Fig. 9:**
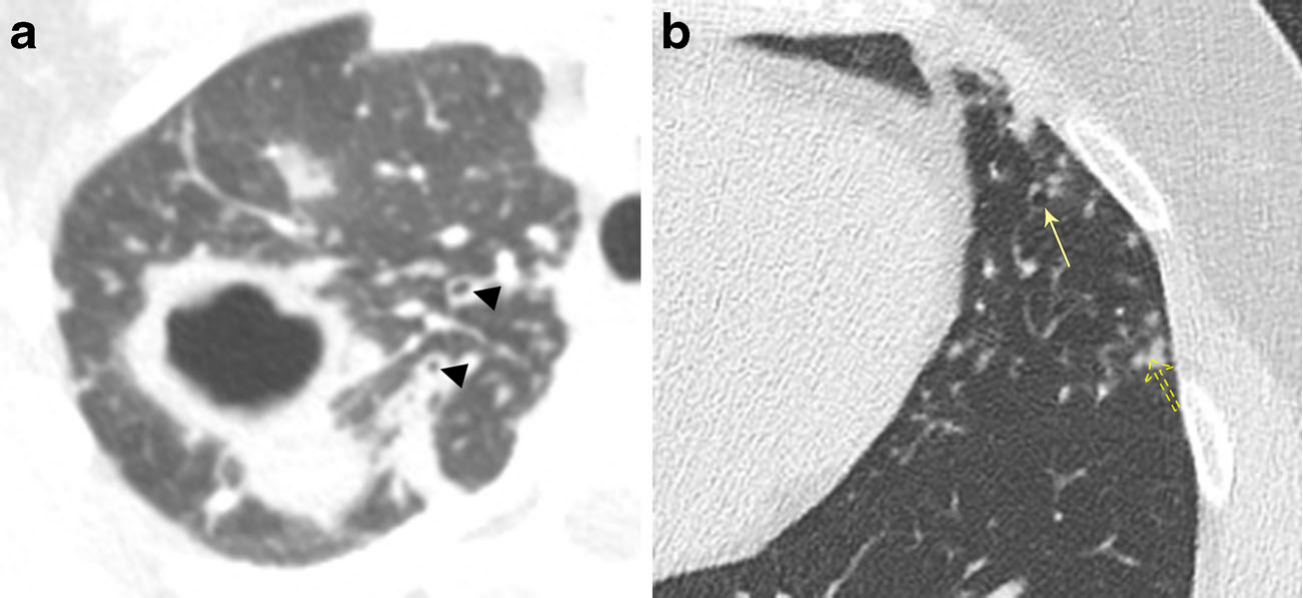
Bronchiectasis due to *Mycobacterial avium complex* (MAC) infection. **a** Axial CT imaging demonstrates an upper lobe-predominant cavitary process typical of the classic form of MAC infection with associated areas of bronchial wall thickening and bronchiectasis (*arrowhead*). **b** Axial CT imaging demonstrates the nonclassical form of MAC infection, also known as Lady Windermere syndrome, characterised by mild to moderate cylindrical bronchiectasis (*solid arrow*) typically affecting the anterior basal right middle lobe and lingula, often with associated ill-defined tree-in-bud or centrilobular nodules (*dashed arrows*)

MAC infection causes different patterns of airspace disease depending on the immune status, behaviour of the patient, and source of infection. Patients typically present with an insidious, often chronic, cough typically productive of purulent sputum. In the classic form, often seen in elderly men with underlying COPD or alcoholism, there is an upper lobe-predominant airspace process characterised by cavitary and ill-defined nodular lesions that mimic tuberculosis infection. Bronchiectasis develops secondary to apical scarring and fibrosis or direct granulomatous damage to the airway (Fig. 9a). The non-classical form of MAC infection, also known as Lady Windermere syndrome, is most commonly seen in elderly women over the age of 60 who actively suppress their cough reflex. Imaging is characterised by mild to moderate cylindrical bronchiectasis typically affecting the anterior basal right middle lobe and lingula, often with associated ill-defined tree-in-bud or centrilobular nodules (Fig. 9b). Atelectasis and scarring of the right middle lobe and lingula may also be present [25, 26]. A third form of MAC infection, also known as hot tub lung, occurs in healthy individuals exposed to aerosolised MAC and will not be reviewed in this article. Management of MAC infection is difficult, often requiring multidrug therapy with treatment regimens lasting at least a year in duration, emphasising the importance of imaging in the early detection and diagnosis of these two presentations. Imaging findings are a particularly important part of the diagnosis for these patients as MAC tends to be difficult to isolate/culture.

#### Bronchiectasis due to allergic bronchopulmonary aspergillosis (ABPA)

**Fig. 10:**
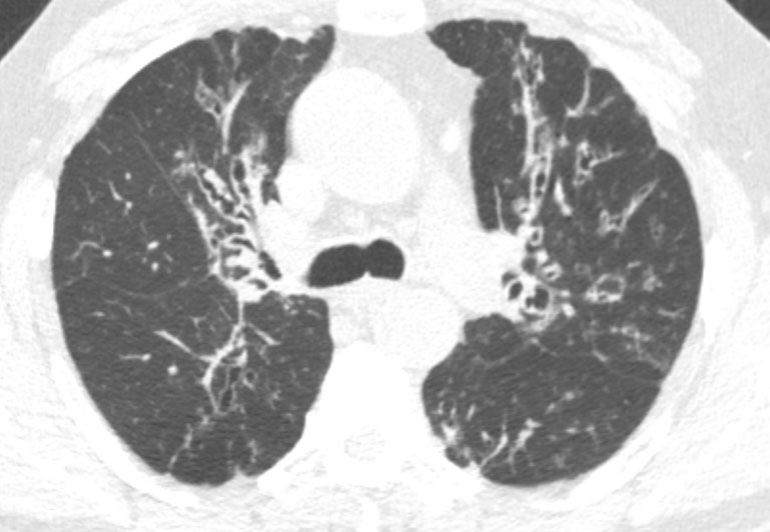
Bronchiectasis due to allergic bronchopulmonary aspergillosis (ABPA). Axial CT image demonstrates the multifocal areas of central bronchiectasis with upper lobe predominance with adjacent areas of atelectasis, ground-glass opacity, and centrilobular nodules

This entity is characterised by a hypersensitivity reaction towards the fungal organism *Aspergillus* growing noninvasively in the lumen of the bronchi. This condition typically occurs in patients with long-standing asthma and is often diagnosed before the age of 40. Imaging typically demonstrates multifocal areas of central bronchiectasis with upper lobe predominance (Fig. 10). High-density mucoid impaction is common, often demonstrating the “finger-in-glove” sign. Other findings include migratory areas of consolidation, atelectasis, ground-glass opacities, mosaic attenuation, and centrilobular nodules [25–27]. Patients with ABPA usually present with recurrent asthma exacerbations, low-grade fever, malaise, cough, and sputum production with mucous plugs [26, 28].

#### Bronchiectasis due to recurrent aspiration

**Fig. 11:**
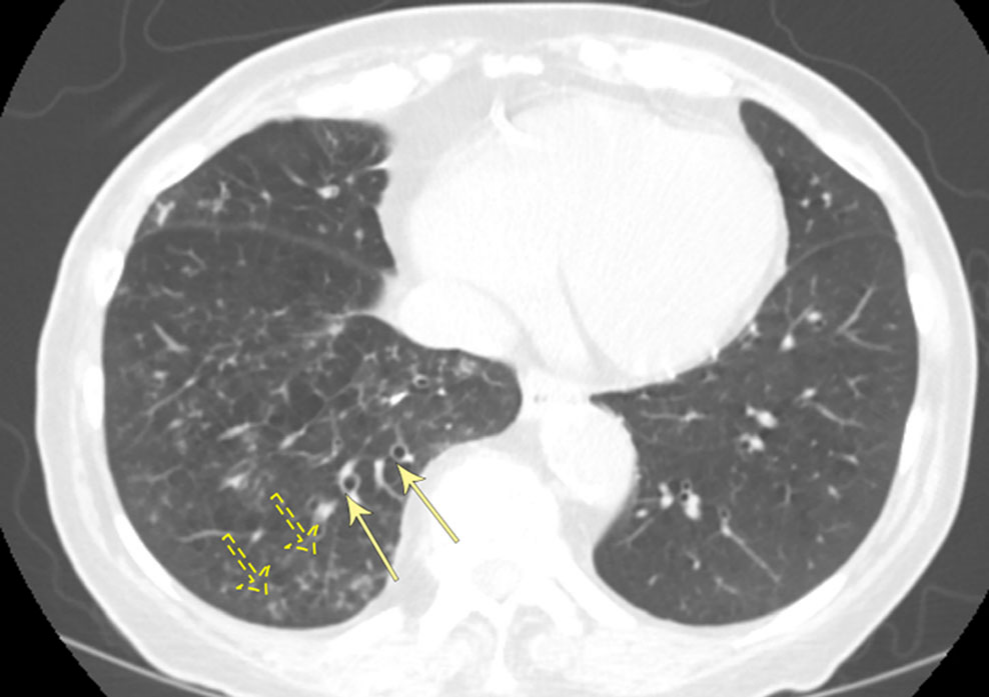
Bronchiectasis due to recurrent aspiration. Axial CT imaging demonstrates sequela of chronic/recurrent aspiration with lower lobe-predominant bronchiectasis (*solid arrows*) and multiple ill-defined centrilobular and tree-in-bud nodules in the dependent portion of the right lower lobe (*dashed arrows*)

Recurrent aspiration with subsequent infection/inflammation causes both direct and indirect damage to the airways and can lead to peripheral and dependent lower lung-predominant bronchiectasis. In the setting of superimposed acute or subacute aspiration, findings consistent with endobronchial inflammation are often seen, including ill-defined centrilobular and tree-in-bud nodules in the dependent portions of the lungs (Fig. 11). Other associated findings include bronchial wall thickening and aspirated fluid contents within the trachea or bronchi [24, 26].

#### Bronchiectasis due to usual interstitial pneumonia (UIP)

**Fig. 12:**
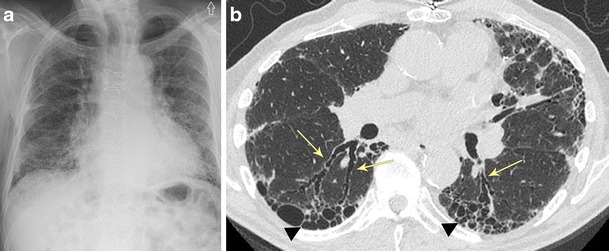
Bronchiectasis due to usual interstitial pneumonia (UIP). **a** Frontal chest x-ray showing prominent coarse interstitial and reticular opacities with a peripheral and basilar predominance, findings consistent with a UIP pattern of fibrosis. **b** Axial CT imaging demonstrating the basilar-predominant varicoid traction bronchiectasis (*arrows*) associated with honeycombing (*arrowhead*) and subpleural reticulation

UIP refers to a specific pattern of interstitial fibrotic lung disease that demonstrates basilar- and peripheral-predominant coarse reticular opacities that extend to the subpleural surface with basilar-predominant honeycombing (Fig. 12a and b). A varicoid-type basilar-predominant bronchiectasis is typically present in end-stage pulmonary fibrosis as the fibrotic lung pulls (or exerts traction on) the adjacent bronchus leading to irreversible dilatation of the bronchial lumen (referred to as traction bronchiectasis) [29]. Associated findings include borderline enlarged thoracic lymphadenopathy, pulmonary parenchymal distortion, and lobar volume loss. Clinically, patients present with slow-onset exertional dyspnoea and nonproductive cough.

#### Bronchiectasis due to cystic fibrosis

**Fig. 13:**
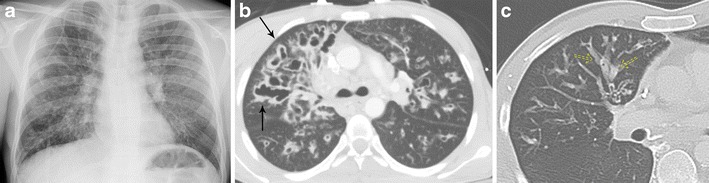
Bronchiectasis due to cystic fibrosis. **a** Frontal chest x-ray demonstrating upper lobe prominent course interstitial markings, patchy areas of increased airspace opacity, and bronchiectasis involving the lungs bilaterally. **b** Axial CT imaging demonstrating upper lobe-predominant cystic and varicoid bronchiectasis and bronchial wall thickening (*solid arrow*). **c** Axial CT imaging in another patient demonstrates bronchiectasis and mucoid impaction (*dashed arrow*) with “finger-in-glove” sign

Cystic fibrosis is an autosomal-recessive condition that results in abnormal chloride transport, primarily affecting the lungs and the pancreas. This condition results in viscous secretions that are difficult to clear, leading to decreased mucus clearance of the airways and subsequent airway obstruction, recurrent infections, and airway damage, which progress over time. CT imaging is sensitive to both the early and late stages of cystic fibrosis. Initially, bronchial wall thickening and peribronchial interstitial prominence are seen [24]. As the disease progresses, upper lobe-predominant bronchiectasis develops, increasing in severity over time (Fig. 13a–c) [25]. Mucoid impaction is often present with associated consolidation/atelectasis. Other typical findings include hyperinflation, mosaic attenuation, bronchiolitis, lymphadenopathy, and pulmonary artery enlargement [30, 31]. Symptoms typically present in childhood with recurrent upper respiratory infection, cough, wheezing, and dyspnoea.

### Obstruction/stenosis

#### Bronchial obstruction due to foreign body

**Fig. 14:**
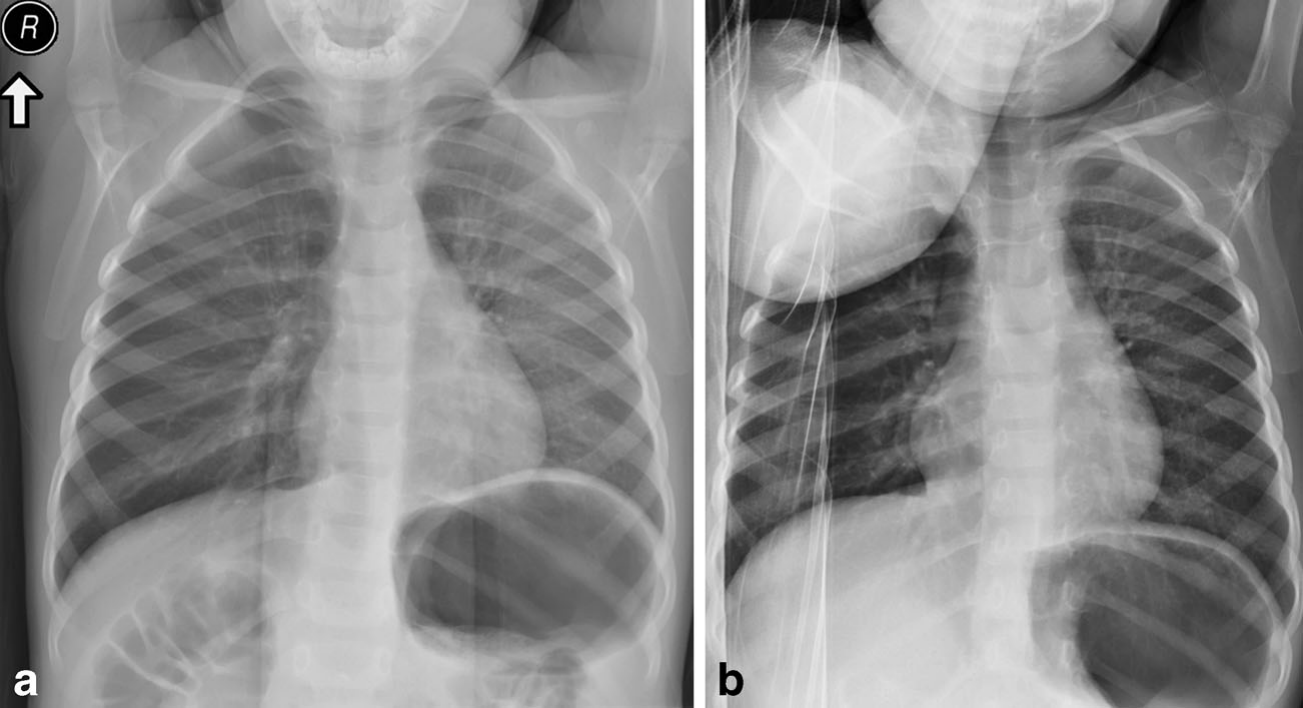
Obstruction due to foreign body. Frontal (**a**) and right lateral decubitus (**b**) images demonstrating mild hyperlucency of the right lung on frontal imaging and lack of volume loss on decubitus imaging, consistent with foreign body obstruction on the right

Foreign body aspiration can potentially lead to partial or complete obstruction of the bronchial tree. It is particularly common in young children but can happen at any age. Plain film imaging, while often negative, may demonstrate unilateral hyperinflation if the object acts as a ball valve or collapse if the foreign body is completely obstructive [32]. Inspiratory/expiratory comparison imaging or decubitus views can be useful complementary studies to identify the affected side, typically demonstrating lack of volume change on the side of obstruction (Fig. 14a and b). CT imaging can be used to identify the location of the foreign body, guide intervention, or assess for retained foreign body post intervention [33]. Due to the wider lumen and more vertical angle of the right main bronchus as compared to the left, foreign body aspiration tends to favour the right lung, commonly involving the gravity-dependent portions of the right middle lobe and right lower lobe. Symptoms commonly include coughing, choking, and wheezing.

#### Bronchial obstruction due to acute or subacute aspiration

**Fig. 15:**
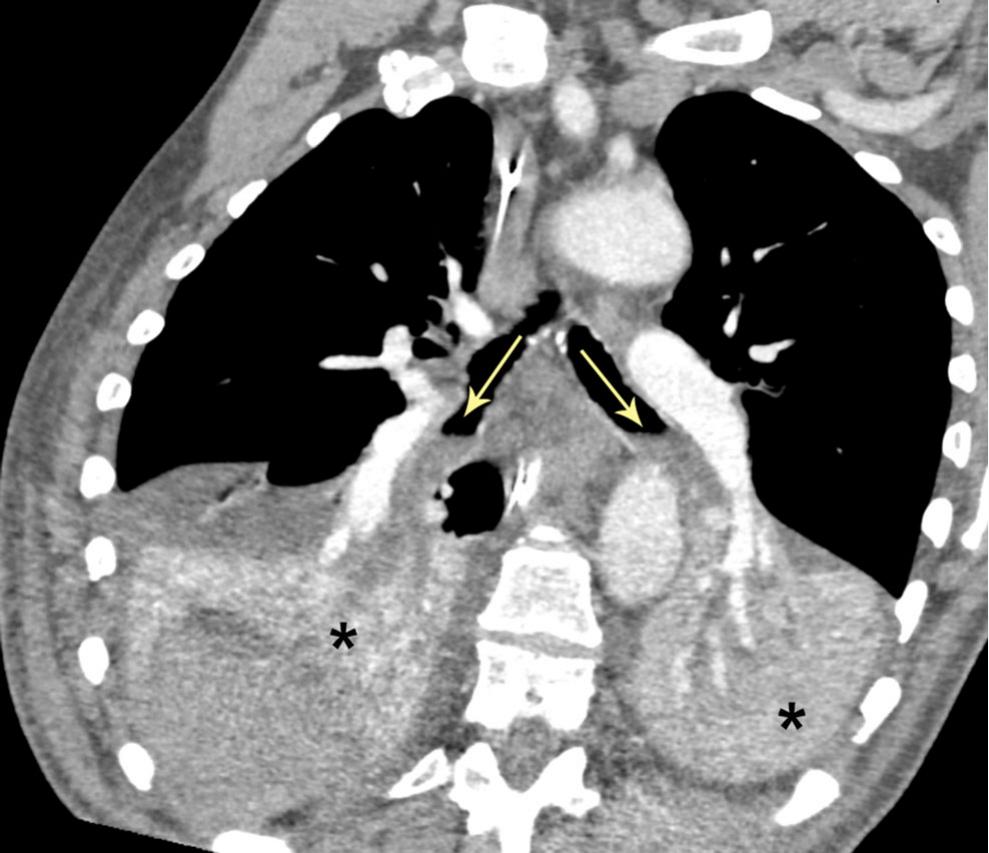
Bronchial obstruction due to acute aspiration. Double oblique CT image of an acute aspiration event. Note the layering low-density fluid in the bilateral lower lobe bronchi (*arrows*) causing complete collapse of the bilateral lower lobes (*)

Acute aspiration is a common cause of bronchial obstruction. Airway involvement is typically gravity dependent. In an upright patient, obstruction typically involves the bilateral lower lobes, right middle lobe, and lingula, whereas in a supine patient obstruction usually involves the posterior aspects of the bilateral upper lobes and superior segments of the lower lobes. Imaging findings are mixed, ranging from interstitial and airway inflammation to areas of consolidation (Fig. 15). Endobronchial material and tree-in-bud and centrilobular nodules are often present [34, 35]. Patients can be asymptomatic or present with coughing, wheezing, tachypnoea, fever, and/or purulent sputum.

#### Bronchial obstruction due to tracheobronchomalacia and excessive dynamic airway collapse

**Fig. 16:**
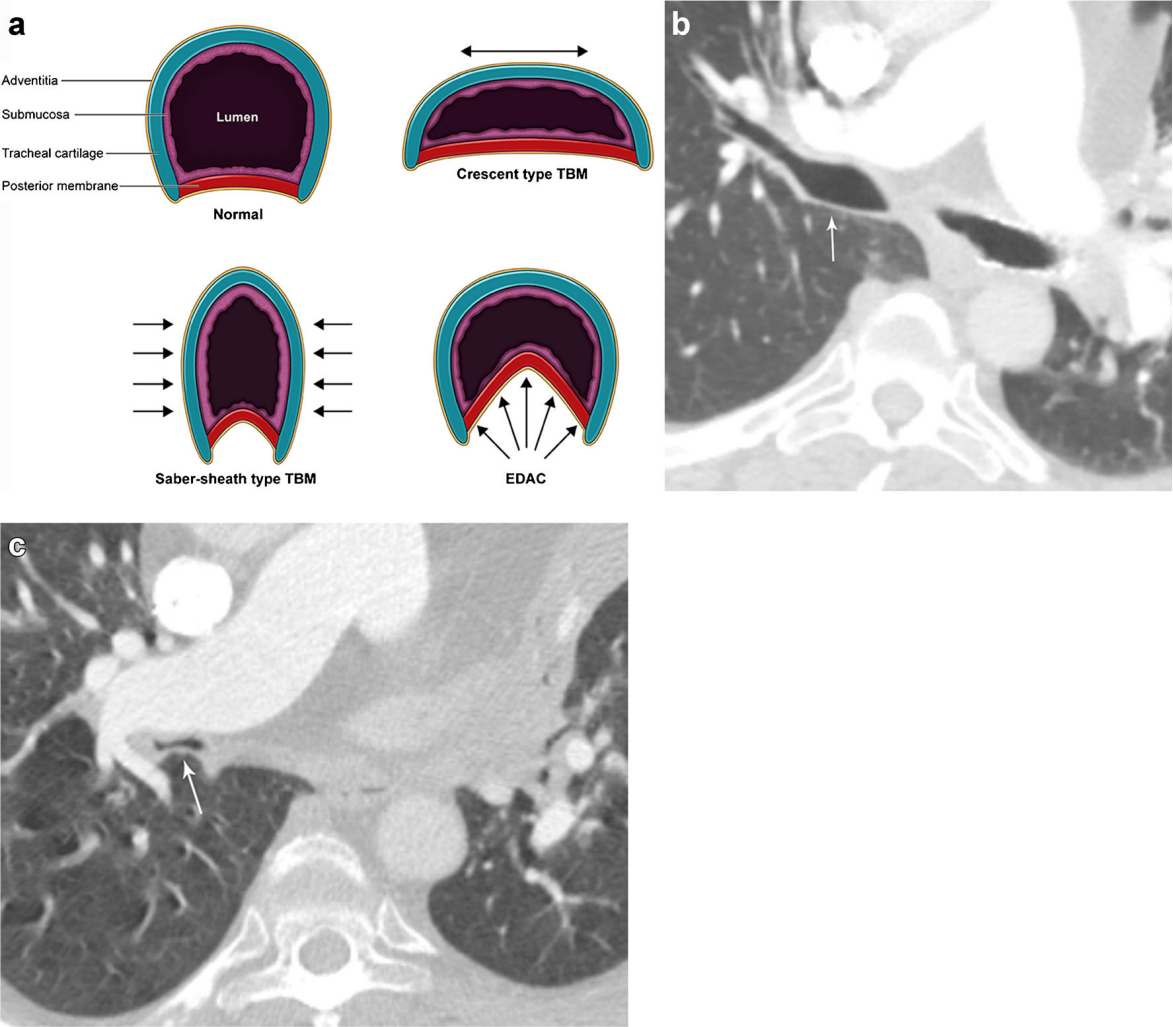
Bronchial obstruction due to tracheobronchomalacia and excessive dynamic airway collapse: **a** Schematic representation of airway collapse in tracheobronchomalacia (TBM) and excessive dynamic airway collapse (EDAC). Axial CT images of a patient with TBM demonstrating a normal-calibre right main bronchus during inspiration (**b**), which collapses with expiratory imaging (**c**). Note that the left main bronchus has been stented to prevent collapse

Tracheobronchomalacia (TBM) refers to weakening of the cartilage support of the bronchus or trachea, typically involving the anterior and/or lateral walls, resulting in excessive airway collapse during expiration. Both congenital and acquired variants of TBM exist with acquired forms arising secondary to chronic inflammation, chronic infection, and damage during intubation [36]. Excessive dynamic airway collapse (EDAC) also results in narrowing of the airway; however the collapse is due to laxity of the posterior longitudinal elastic fibres with normal cartilaginous rings [37]. These two can be identified as distinct entities on dynamic CT imaging with respiratory manoeuvres. In TBM, the cartilaginous portions of the walls collapse with anterior involvement decreasing the anterior-posterior diameter (crescent-shaped TBM) and lateral involvement decreasing the transverse diameter (saber sheath TBM) (Fig. 16a). In EDAC, only the posterior membrane becomes lax, bulging anteriorly while the rest of the airway wall remains intact (also known as the “frown sign”) (Fig. 16a) [6]. In both cases, airway collapse of greater than 70% between end-inspiratory and end-expiratory imaging is diagnostic (Fig. 16b and c) [6, 38]. Both conditions typically result in dyspnoea, increased sputum production, and infection [6].

#### Bronchial obstruction due to neoplasm

**Fig. 17:**
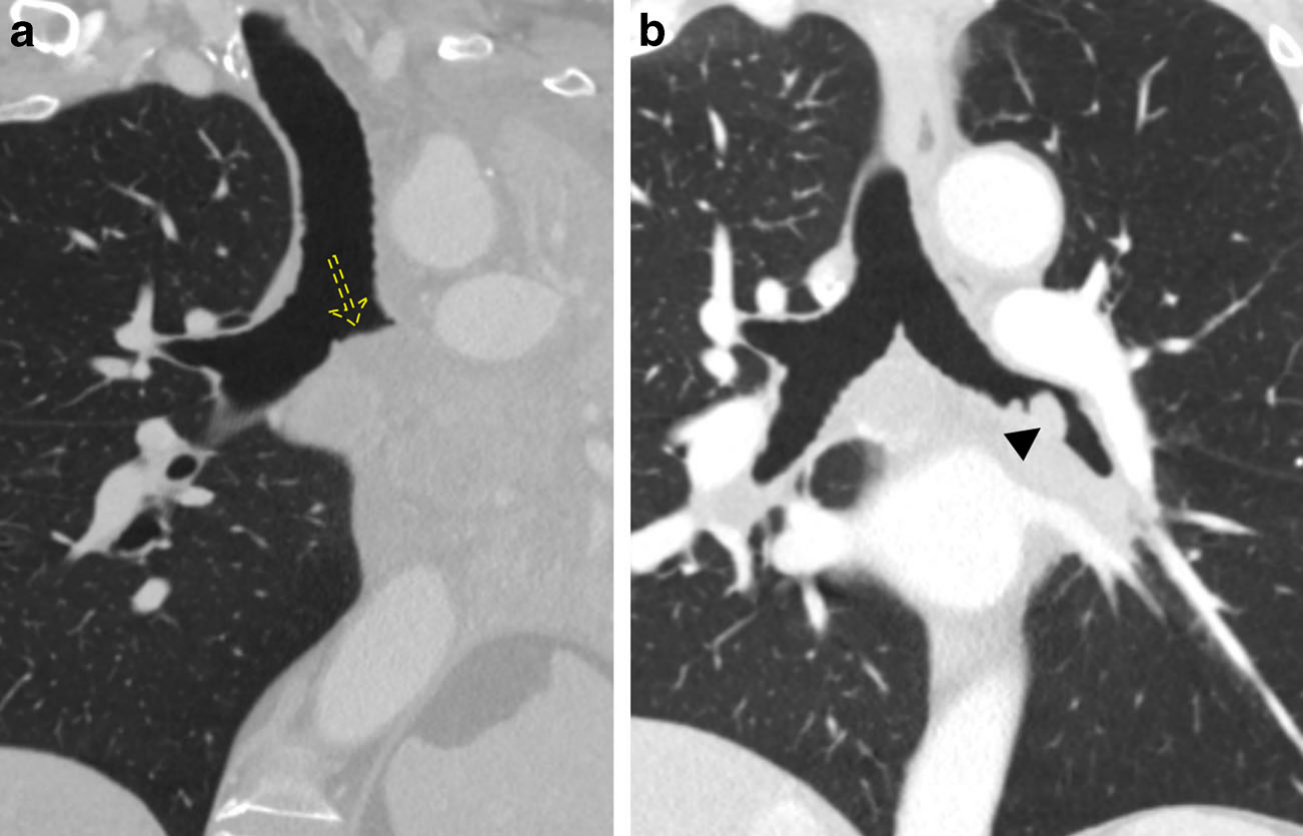
Bronchial obstruction due to neoplasm. Coronal CT imaging of metastatic renal carcinoma before (**a**) and after (**b**) chemotherapy. Note that prior to chemotherapy (**a**) there is obstruction of the left main bronchus with an air fluid level (*dashed arrow*) and complete collapse of the left lung. After several rounds of chemotherapy (**b**) the endoluminal metastatic renal cell carcinoma is now seen (*arrowhead*) and is no longer obstructive with interval re-expansion of the left lung

Neoplasms, both benign and malignant, are common causes of bronchial obstruction. Occlusion can be secondary to endoluminal growth or extrinsic compression. One of the most commonly encountered bronchial neoplasms is carcinoid tumour, a neuroendocrine neoplasm that favours the central bronchi [39]. Imaging typically shows a well-defined, round or oval endoluminal perihilar enhancing mass, often with calcification. Primary lung malignancy, especially centrally located neoplasms such as small cell lung cancer, and metastatic disease are common causes of bronchial obstruction. While these lesions typically cause extrinsic compression or direct invasion of the bronchus, they can also have endoluminal growth. Post-obstructive atelectasis or pneumonia commonly occurs with all forms of bronchial obstruction including neoplasms (Fig. 17a and b). Patients often present with cough, haemoptysis, wheezing, and recurrent pneumonia. In the setting of malignancy, constitutional symptoms of weight loss, malaise, and night sweats may also be present.

#### Bronchial obstruction due to granulomatous disease

**Fig. 18:**
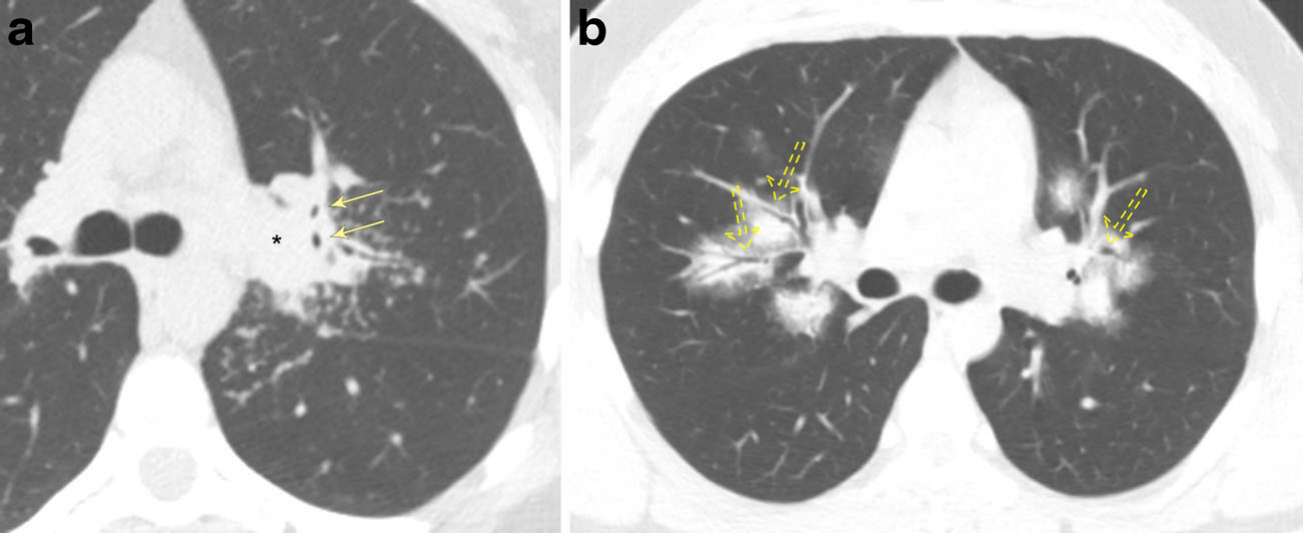
Bronchial obstruction due to granulomatous disease. **a** Axial CT image of sarcoidosis demonstrating perihilar lymphadenopathy (*) and pulmonary granulomas causing extrinsic compression of several left upper lobe segmental bronchi (*arrows*). **b** Axial CT imaging of granulomatosis with polyangitis demonstrates infiltrative changes of the pulmonary parenchyma in a perihilar/bronchovascular distribution causing narrowing of the associated bronchi in a bilateral upper lobe predominance with sparing of the apices. The associated ground-glass opacities suggest the presence of haemorrhage

This is a broad group of pulmonary conditions, which are characterised by the formation of granulomas. Obstruction can be from endoluminal granuloma formation or secondary to extrinsic compression. Common noninfectious aetiologies include sarcoidosis, silicosis/pneumoconioses, Langerhans cell histiocytosis, granulomatosis with polyangitis, and hypersensitivity pneumonitis. Infectious aetiologies include tuberculosis, histoplasmosis, coccidioidomycosis, and cryptococcosis. Imaging often reveals pulmonary nodules and lymphadenopathy, some of which may demonstrate calcification [40–42]. Granulomatous disease tends to be upper lobe predominant and bilateral (Fig. 18a and b). While some patients are asymptomatic, common symptoms include fatigue, weight loss, fever, malaise, and night sweats.

#### Bronchial obstruction due to a broncholith

**Fig. 19:**
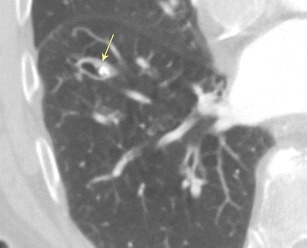
Bronchial obstruction due to a broncholith. CT image demonstrating calcified material (*arrow*) within the right lower lobe bronchus

Broncholiths can arise from a concretion (hard substance formed around an inorganic centre) or from erosion of a calcified pulmonary nodule into an adjacent bronchus. These lesions can be partially or completely obstructive. Concretions typically are 2–15 mm irregularly shaped endoluminal calcifications with angled margins following the contour of the bronchus, whereas eroded calcified nodules are often round or oval in shape (Fig. 19) [43]. Associated findings commonly include mucoid impaction, post-obstructive atelectasis, air-trapping, and distal bronchiectasis [44]. Broncholith formation favours the right middle lobe and the bilateral upper lobes. Patients may be asymptomatic or present with a nonproductive cough, haemoptysis, or recurrent infections.

#### Bronchial obstruction due to asthma

**Fig. 20:**
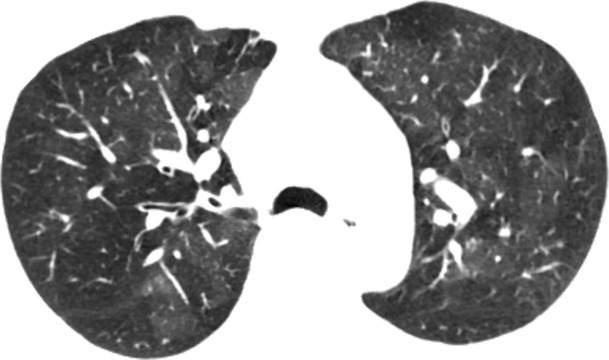
Bronchial obstruction due to asthma. Axial CT image in a patient during an asthma exacerbation demonstrating a mosaic attenuation pattern of the pulmonary parenchyma consistent with air-trapping

Asthma is a chronic inflammatory condition of the airways typified by reversible airway obstruction and at least partially reversible inflammation. In patients with severe or chronic recurrent asthmatic symptoms, the hyperactive airways demonstrate chronic inflammation, bronchial oedema, and wall thickening. Plain film and CT imaging in asthma patients is often unremarkable. If severe and/or chronic, hyperinflation, mosaic attenuation, bronchial wall thickening, and bronchiectasis can be seen (Fig. 20). Patients classically present with episodic wheezing, cough, dyspnoea, and chest tightness.

## Summary

Bronchial disorders are both congenital and acquired in aetiology. Acquired bronchial disorders can often be subdivided into one of three categories based on imaging findings: wall thickening, dilatation/bronchiectasis, and obstruction/stenosis. It is the role of the radiologist to recognise these imaging patterns and provide an accurate differential diagnosis tailored to a patient’s clinical situation to assist the ordering physician in patient management.
